# Diagnostic performance of MRI, SPECT, and PET in detecting renal cell carcinoma: a systematic review and meta-analysis

**DOI:** 10.1186/s12885-022-09239-3

**Published:** 2022-02-11

**Authors:** Qihua Yin, Huiting Xu, Yanqi Zhong, Jianming Ni, Shudong Hu

**Affiliations:** 1 Department of Radiology, Wuxi No. 2 People’s Hospital Affiliated to Nanjing Medical University, Address: No. 68, Zhongshan Rd., Wuxi, 214002 Jiangsu Province China; 2 Department of Radiology, Affiliated Hospital of Jiangnan University, No. 1000, Hefeng Road, Wuxi, 214122 China; 3 Shanghai Key Laboratory of Molecular Imaging, Shanghai University of Medicine and Health Sciences, Shanghai, 201318 China

**Keywords:** MRI, SPECT, PET, Renal cell carcinoma, Diagnostic performance, Meta-analysis

## Abstract

**Background:**

Renal cell carcinoma (RCC) is one of the most common malignancies worldwide. Noninvasive imaging techniques, such as magnetic resonance imaging (MRI), single photon emission computed tomography (SPECT), and positron emission tomography (PET), have been involved in increasing evolution to detect RCC. This meta-analysis aims to compare to compare the performance of MRI, SPECT, and PET in the detection of RCC in humans, and to provide evidence for decision-making in terms of further research and clinical settings.

**Methods:**

Electronic databases including PubMed, Web of Science, Embase, and Cochrane Library were systemically searched. The keywords such as “magnetic resonance imaging”, “MRI”, “single-photon emission computed tomography”, “SPECT”, “positron emission tomography”, “PET”, “renal cell carcinoma” were used for the search. Studies concerning MRI, SPECT, and PET for the detection of RCC were included. Pooled sensitivity, specificity, and the area under the summary receiver operating characteristic (SROC) curve (AUC), etc. were calculated.

**Results:**

A total of 44 articles were finally detected for inclusion in this study. The pooled sensitivities of MRI, ^18^F-FDG PET and ^18^F-FDG PET/CT were 0.80, 0.83, and 0.89, respectively. Their respective overall specificities were 0.90, 0.86, and 0.88. The pooled sensitivity and specificity of MRI studies at 1.5 T were 0.86 and 0.94, respectively. With respect to prospective PET studies, the pooled sensitivity, specificity and AUC were 0.90, 0.93 and 0.97, respectively. In the detection of primary RCC, PET studies manifested a pooled sensitivity, specificity, and AUC of 0.77, 0.80, and 0.84, respectively. The pooled sensitivity, specificity, and AUC of PET/CT studies in detecting primary RCC were 0.80, 0.85, and 0.89.

**Conclusion:**

Our study manifests that MRI and PET/CT present better diagnostic value for the detection of RCC in comparison with PET. MRI is superior in the diagnosis of primary RCC.

## Introduction

Renal cancer is one of the most frequently diagnosed cancers worldwide, which ranks the 6th most frequently confirmed malignant tumor in men and the 8th in women [1]. 90% of all renal malignant tumors tend to be renal cell carcinoma (RCC) on a histopathological basis [2, 3]. There are three major histological subtypes of renal cell carcinoma: clear cell RCC, papillary RCC, and chromophobe RCC [4]. It is manifested that over one-half of patients with renal cell carcinoma are asymptomatic [5]. Approximately 33 to 50% of suspected patients are diagnosed with metastatic diseases at the time of initial detection, furthermore, 20 to 40% of patients with confirmed RCC progress to metastatic diseases even after surgical resection [6, 7]. Consequently, timely and accurate detection of the early stage and advanced stage of the disease is of great significance. Partial or radical nephrectomy is still the gold standard for the treatment of renal tumors, no significant benefit have been proved regarding RCC adjuvant therapies [5].

Biopsy diagnosis is still the gold standard for confirmation of RCC although it is an invasive modality that may result in unnecessary adverse outcomes [8]. Various noninvasive imaging approaches are commonly employed in the detection of RCC [9]. For decades, ultrasound (US) has been used as one of the first-line modalities for diagnostic imaging of patients with renal lesions due to its cost-effective nature, however, the efficacy of US is not satisfactory especially in patients with suspected malignancies [9]. Although computed tomography (CT) has been utilized as the confirmative standard for RCC imaging for decades, it manifested poor performance in differentiation among solid masses, fat-poor angiomyolipoma (AML), and oncocytoma [10, 11]. Compared to CT, magnetic resonance imaging (MRI) plays an increasingly important role in the diagnosis and restaging of RCC, particularly in patients with unclear results, allergic reactions, pregnancies, as it has no ionizing radiation exposure and superior soft tissue resolution [12, 13]. Although contrast-enhanced MRI performed better than diffusion-weighted (DW) MRI for the diagnosis of RCC, patients who have renal dysfunction are at risk for nephrogenic systemic fibrosis or contrast material–induced nephropathy [14]. In recent years, targeted imaging approaches have made great progression in the diagnosis of RCC. Single photon emission computed tomography-computed tomography (SPECT) imaging is used to differentiate RCC and detect metastases in renal cancer [15, 16]. Furthermore, positron emission tomography (PET) imaging utilizing ^18^F-fluoro-deoxy-glucose (FDG) and other tracers (^124^I-girentuximab, ^68^Ga-DOTATOC, ^11^C-acetate, ^18^F-fluoride) has been studied as diagnostic biomarkers in RCC [17–22]. Especially, PET plays an important role in the detection of recurrent or metastatic RCC [22, 23]. Furthermore, specific European Association of Nuclear Medicine (EANM) procedure guidelines have been intended to assist practitioners in performing, interpreting and reporting the results of FDG PET/CT for imaging of patients [24].

A large number of studies have assessed the diagnostic performance of non-invasive approaches in terms of RCC, nevertheless, the results are heterogeneous [15, 18, 22, 23, 25–27]. This study aimed to generate a more comprehensive comparison of the diagnostic performance of MRI, SPECT, and PET in the detection of RCC by conducting a meta-analysis, and subsequently to guide the diagnosis and differentiation of RCC in the field of scientific research and clinical application.

## Materials and methods

The entire process of this study was conducted based on the Preferred Reporting Items for Systematic Reviews and Meta-analysis (PRISMA) [28].

### Search strategy and selection criteria

The electronic databases of PubMed, Web of Science, Embase, and Cochrane Library were comprehensively searched with a publication date from inception to January 31, 2021. Articles in the English language were considered. The following key terms were used for the database research: “magnetic resonance imaging”, “MRI”, “single-photon emission computed tomography”, “SPECT”, “positron emission tomography”, “PET”, “renal cell carcinoma”. Besides, we manually screened the references of the articles included for more potentially eligible studies. The inclusion criteria of studies were as follows: 1) MRI, SPECT, and/or PET were used for the detection of RCC in patients with suspected or confirmed RCC; 2) a reference standard was utilized to assess diagnostic performance; 3) absolute numbers of patients with true positive (TP), false positive (FP), true negative (TN) and false negative (FN) results can be retrieved in the published articles or recalculated based on other parameters (accuracy rate, sensitivity, specificity, positive predictive value (PPV), negative predictive value (NPV), number of all participants) presented in the manuscripts. In case that the studies were undertaken by the same research group, those with the largest sample size or the most complete information were included to avoid duplicates. Articles were excluded if they were case reports, reviews, letters, news, conference abstracts, animal studies, or studies with insufficient data.

Two independent investigators (QY and HX) conducted the process of literature search and study inclusion. Discrepancies were resolved by discussion. If no consensus was reached, a third author (JN) was involved.

### Data extraction and quality assessments

Two researchers (QY and YZ) independently performed the title and abstract screening according to the inclusion criteria. A full-text reading of the literature was conducted for the final inclusion. The following information was extracted from each study: first author’s name, year of publication, study design, type of RCC (primary or recurrent/metastatic), number of patients analyzed, percentage of the male, age of the participants, reference standard, imaging modality and type of radiotracers used in the study, absolute numbers of patients with TP, TN, FP, and FN numbers.

To evaluate the methodological quality of the enrolled studies, we used the Quality Assessment of Diagnostic Accuracy Studies-2 (QUADAS-2) tool. This method contains four main components in terms of participant selection, index test, reference standard, as well as flow and timing, all the components are assessed in terms of risk of bias, and the first three components are also evaluated the concerns of applicability [29].

### Statistical analysis

We calculated pooled sensitivity, specificity, positive likelihood ratio (PLR), negative likelihood ratio (NLR), diagnostic odds ratio (DOR), and the 95% confidence intervals (CIs) and the area under the summary receiver operating characteristic (SROC) curve (AUC). A Cochran Q value and the I^2^ statistic were used to detect the heterogeneity of studies included. I^2^ statistics in the range of 0–25%, 25–50%, 50–75%, and 75–100% were considered to be of insignificant, low, moderate, and high heterogeneity between studies, respectively [30]. Meta-regression was performed to investigate the possible source of heterogeneity between the included studies. A Deeks’ method was introduced to statistically test the asymmetry of the funnel plot and detect publication bias. We conducted sensitivity analysis to evaluate the impacts of one single study on the overall outcomes. All statistical analyses were processed on the study basis using the Stata 15.0 software and Review Manager 5.3 software. A *p* value < 0.05 was considered to be statistically significant.

## Results

### Study selection and characteristics

**Fig. 1:**
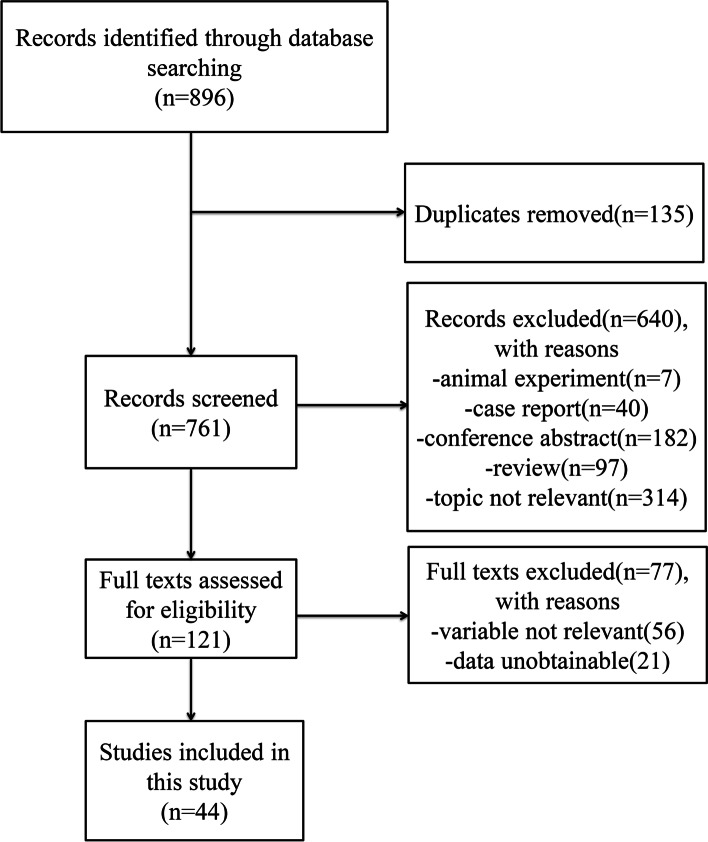
Search results and flow chart of the meta-analysis

**Fig. 2:**
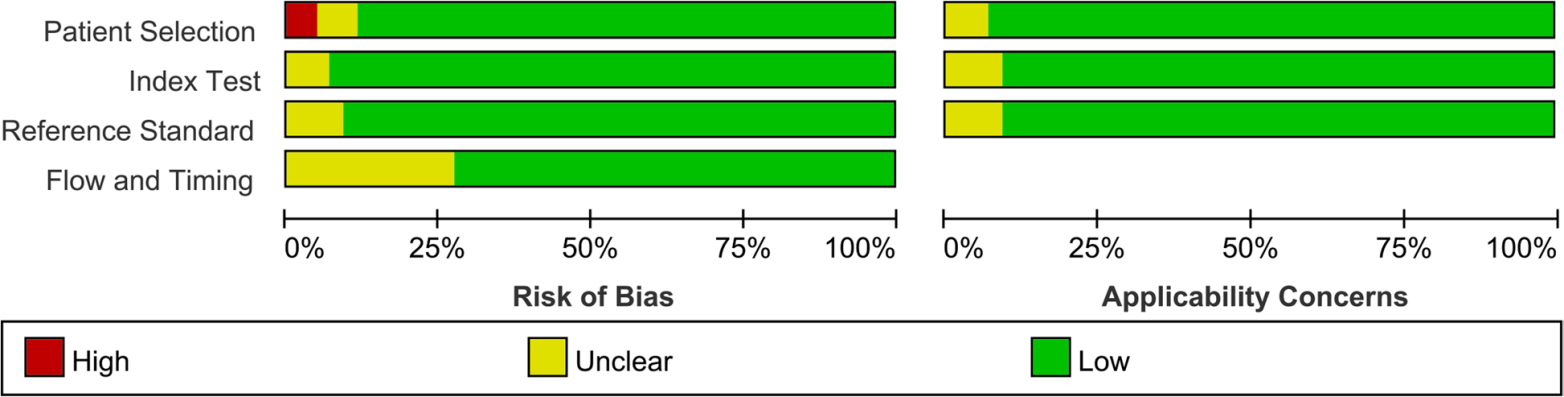
Methodological assessment of studies included on the QUADAS-2 tool

A total number of 896 articles were identified from the online databases. Among them, we excluded 135 duplicates and 640 irrespective studies based on an initial screening of titles and abstracts. After the full text confirmation for eligibility of the remaining 121 articles, 44 articles with 50 studies and 2545 patients were identified for final inclusion in this study. No additional studies were found through reference screening of the included papers. Figure 1 shows the flow of the database search and literature selection process. Detailed characteristics of studies included were shown in Table 1. The results of the quality evaluation of the included studies manifested that the high quality of the included studies (Fig. 2).

**Table 1:** Study characteristics

Name of the first author	Year of publication	Study design	Population	Type of RCC	Reference test	No. of Patients analysed	Male, No. (%)	Age (SD or IQR)	Modalities	Image analysis	Tracers
Adams	2019	Prospective	Suspected or known RCC	Primary	Histopathology	27	70	61 (14)	MRI	Quantitative	–
Aide	2003	Prospective	Suspected RCC	Primary	Histopathology	35	60	60 (14)	PET	Qualitative	^18^F-FDG
Alongi	2016	Prospective	Suspected or known RCC	Primary	Histopathology and/or other imaging	104	69	63 (13)	PET/CT	Qualitative	^18^F-FDG
Aslan	2018	Retrospective	Known RCC	Primary	Histopathology	49	52	62 (14)	MRI	Qualitative	–
Bertagna	2013	Retrospective	Known RCC	Primary	Histopathology	68	72	68 (4)	PET/CT	Qualitative	^18^F-FDG
Chang	2003	Retrospective	Known RCC	Primary	Histopathology	15	47	56 (15)	PET	Qualitative	^18^F-FDG
Chen	2014	Retrospective	Suspected RCC	Primary	Histopathology	35	57	57 (29–77)	MRI	Qualitative	–
Chen	2015	Prospective	Suspected RCC	Primary	Histopathology	71	75	50 (21–78)	MRI	Qualitative	–
Choi	2021	Retrospective	Known RCC	Primary	Histopathology	110	67	NR	MRI	Qualitative	–
Dilhuydy	2006	Retrospective	Suspected or known RCC	Recurrent or metastatic	Histopathology	18	NR	57 (13)	PET/CT	Qualitative	^18^F-FDG
Ding	2016	Retrospective	Suspected or known RCC	Primary	Histopathology	35	58	52 (12)	MRI	Qualitative	–
Divgi	2013	Prospective	Suspected or known RCC	Primary	Histopathology	204	63	56 (12)	PET/CT	Qualitative	^124^I-girentuximab
Fisher	2008	Prospective	Suspected or known RCC	Primary	Histopathology	122	64	58 (32–80)	SPECT	Qualitative	^99m^Tc-EC20
Fuccio	2014	Retrospective	Known RCC	Primary	Histopathology	69	NR	62 (36–86)	PET/CT	Qualitative	^18^F-FDG
Goldberg	1997	Prospective	Suspected or known RCC	Primary	Histopathology	10	57	49 (37–76)	PET	Qualitative	^18^F-FDG
Jadvar	2003	Retrospective	Suspected or known RCC	Primary	Histopathology and clinical follow-up	25	72	NR	PET	Qualitative	^18^F-FDG
Johnson	2019	Retrospective	Suspected or known RCC	Primary	Histopathology	57	NR	62 (15)	MRI	Qualitative	–
Kang	2004	Prospective	Suspected or known RCC	Primary	Histopathology	17	74	59 (28–79)	PET	Qualitative	^18^F-FDG
Kumar	2010	Retrospective	Known RCC	Primary	Histopathology and clinical follow-up and conventional imaging finding	103	87	57 (12)	PET/CT	Qualitative	^18^F-FDG
Kwon	2015	Prospective	Suspected or known RCC	Primary	Histopathology and clinical follow-up	73	60	52 (28–71)	MRI	Qualitative	–
Li	2020	Retrospective	Known RCC	Primary	Histopathology	127	78	56 (12)	MRI	Quantitative	–
Lima	2020	Retrospective	Known RCC	Primary	Histopathology	47	55	61 (12)	MRI	Qualitative	–
Lyu	2018	Retrospective	Suspected metastatic RCC	Recurrent or metastatic	Histopathology	35	54	55 (15)	MRI	Qualitative	–
Majhail	2003	Retrospective	Suspected metastatic RCC	Recurrent or metastatic	Histopathology	36	79	63 (45–82)	PET	Qualitative	^18^F-FDG
Miyakita	2002	Retrospective	Known RCC	Primary	Histopathology	19	79	57 (10)	PET	Qualitative	^18^F-FDG
Muselaers	2013	Retrospective	Suspected RCC	Primary	Histopathology	29	41	64 (8)	SPECT	Qualitative	^111^In-Girentuximab
Nakamoto	2019	Retrospective	Suspected or known recurrent RCC	Primary	Histopathology	25	76	64 (38–86)	PET/CT	Qualitative	^68^Ga-DOTATOC/^18^F-FDG
Nakatani	2011	Retrospective	Suspected recurrent RCC	Primary	Histopathology and clinical follow-up	28	75	63 (45–78)	PET	Qualitative	^18^F-FDG
Oyama	2014	Prospective	Suspected RCC	Primary	Histopathology	34	NR	67 (38–87)	PET	Qualitative	^18^F-FDG/^11^C-acetate
Ozturk	2016	Retrospective	Suspected recurrent or metastatic lesions	Recurrent or metastatic	Histopathology or clinical follow-up	132	68	61 (12)	PET	Qualitative	^18^F-FDG
Ozulker	2011	Prospective	Suspected RCC	Primary	Histopathology	18	44	57 (11)	PET/CT	Qualitative	^18^F-FDG
Park	2009	Retrospective	Suspected recurrent or metastatic lesions	Recurrent or metastatic	Histopathology or by clinical follow-up	63	75	54 (31–76)	PET/CT	Qualitative	^18^F-FDG
Purkayastha	2020	Retrospective	Known RCC	Primary	Histopathology	43	82	64 (38–85)	MRI	Quantitative	–
Ramdave	2001	Prospective	Suspected or known primary RCC	Primary	Histopathology	17	56	61 (32–79)	PET	Qualitative	^18^F-FDG
Safaei	2002	Prospective	Suspected RCC	Primary	Histopathology	36	78	54 (11)	PET	Qualitative	^18^F-FDG
Sharma	2014	Prospective	Suspected metastatic RCC	Recurrent or metastatic	Histopathology	16	75	53 (14)	PET/CT	Qualitative	^18^F-Fluoride
Sheikhbahaei	2017	Prospective	Suspected RCC	Primary	Histopathology	48	73	59 (40–81)	SPECT/CT	Qualitative	^99m^Tc-MIBI
Sistani	2020	Prospective	Known RCC	Primary	Histopathology	31	79	60 (30–83)	SPECT/CT	Qualitative	^99m^Tc-MIBI
Sohaib	2009	Prospective	Suspected metastatic RCC	Recurrent or metastatic	Histopathology	47	70	62 (29–79)	SPECT/MRI	Qualitative	^99m^Tc-MDP
Sun	2020	Retrospective	Known RCC	Primary	Histopathology	45	60	57 (12)	MRI	Quantitative	–
Win	2015	Retrospective	Suspected metastatic RCC	Recurrent or metastatic	Histopathology	315	85	47.5	PET/CT	Qualitative	^18^F-FDG
Wu	2002	Prospective	Suspected metastatic RCC	Recurrent or metastatic	Histopathology	52	67	(46–73)	SPECT/PET	Qualitative	^99m^Tc-MDP/^18^F-FDG
Youssef	2018	Prospective	Suspected metastatic RCC	Recurrent or metastatic	Histopathology	20	65	NR	PET/CT	Qualitative	^18^F-FDG
Zhao	2020	Retrospective	Known RCC	Primary	Histopathology	35	69	61	MRI	Quantitative	–

*RCC* renal cell carcinoma, *SD* standard deviation, *IQR* interquartile range, *MRI* magnetic resonance imaging, *SPECT* single photon emission computed tomography, *PET* positron emission tomography, *NR* not reported

### Diagnostic performance of imaging modalities

**Fig. 3:**
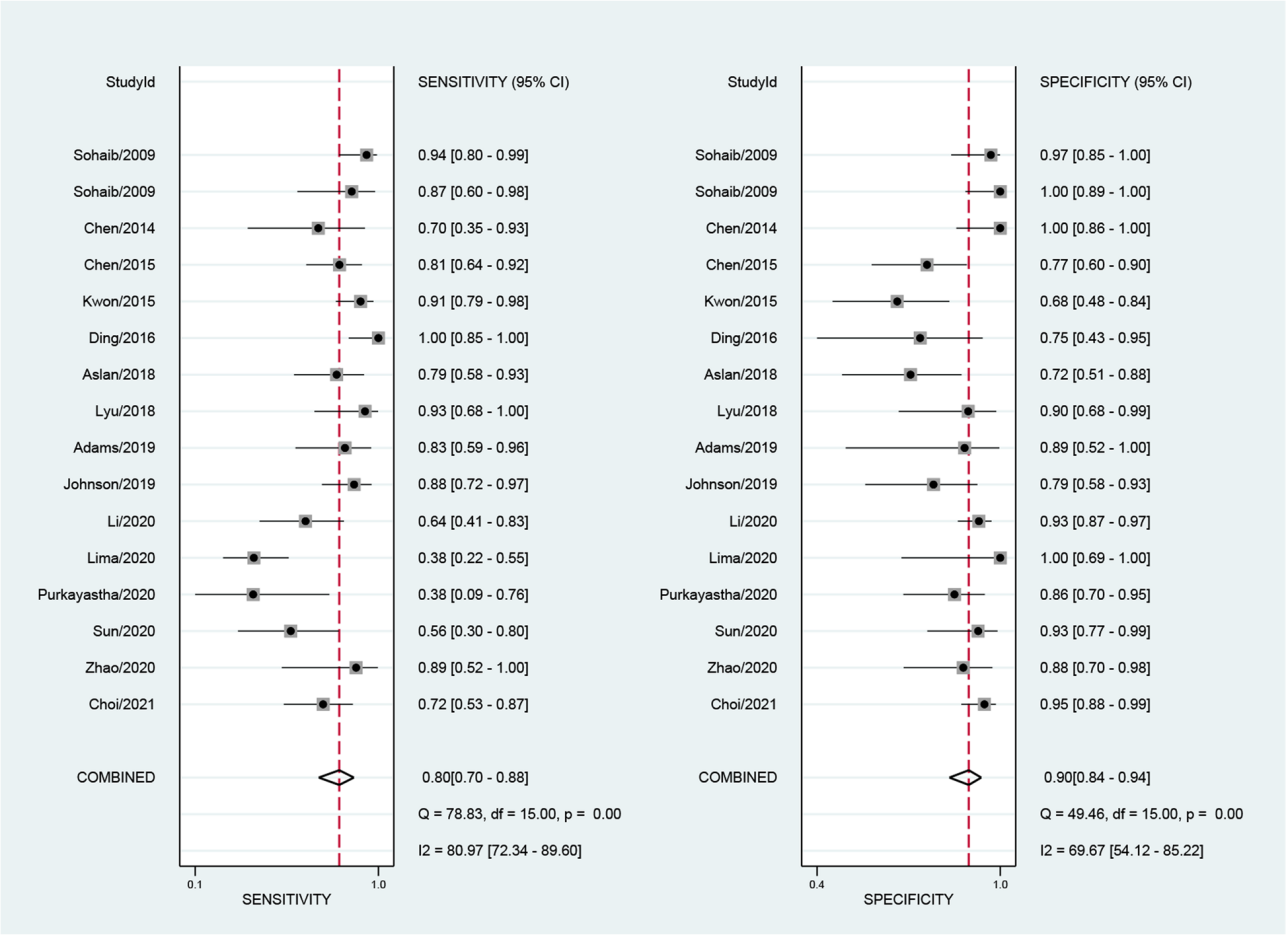
Forest plot for the detection performance of MRI

**Fig. 4:**
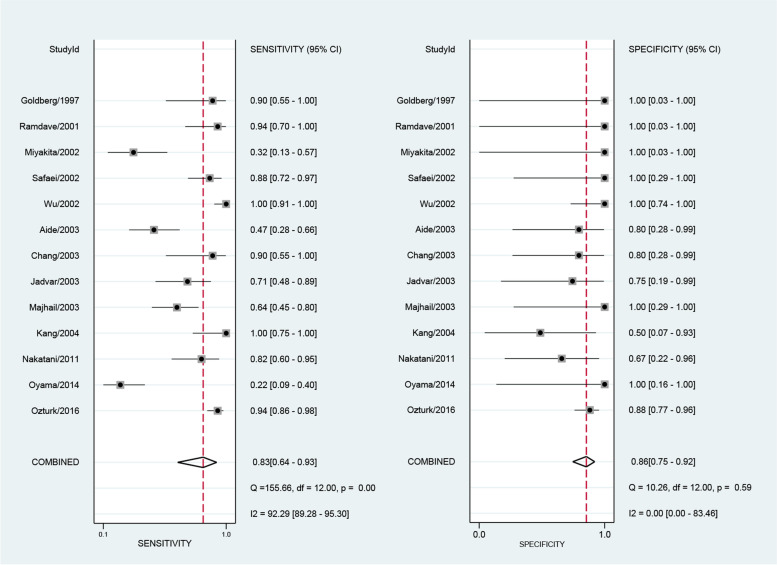
Forest plot for the detection performance of ^18^F-FDG PET

**Fig. 5:**
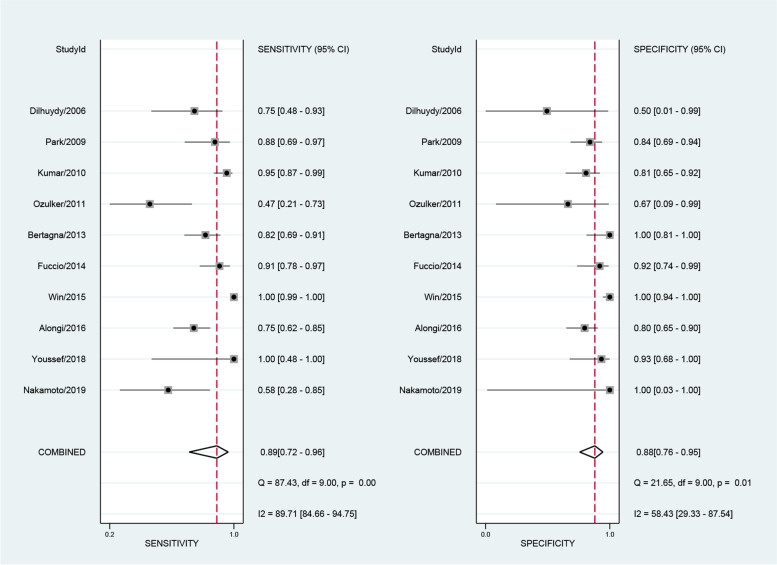
Forest plot for the detection performance of ^18^F-FDG PET/CT

**Fig. 6:**
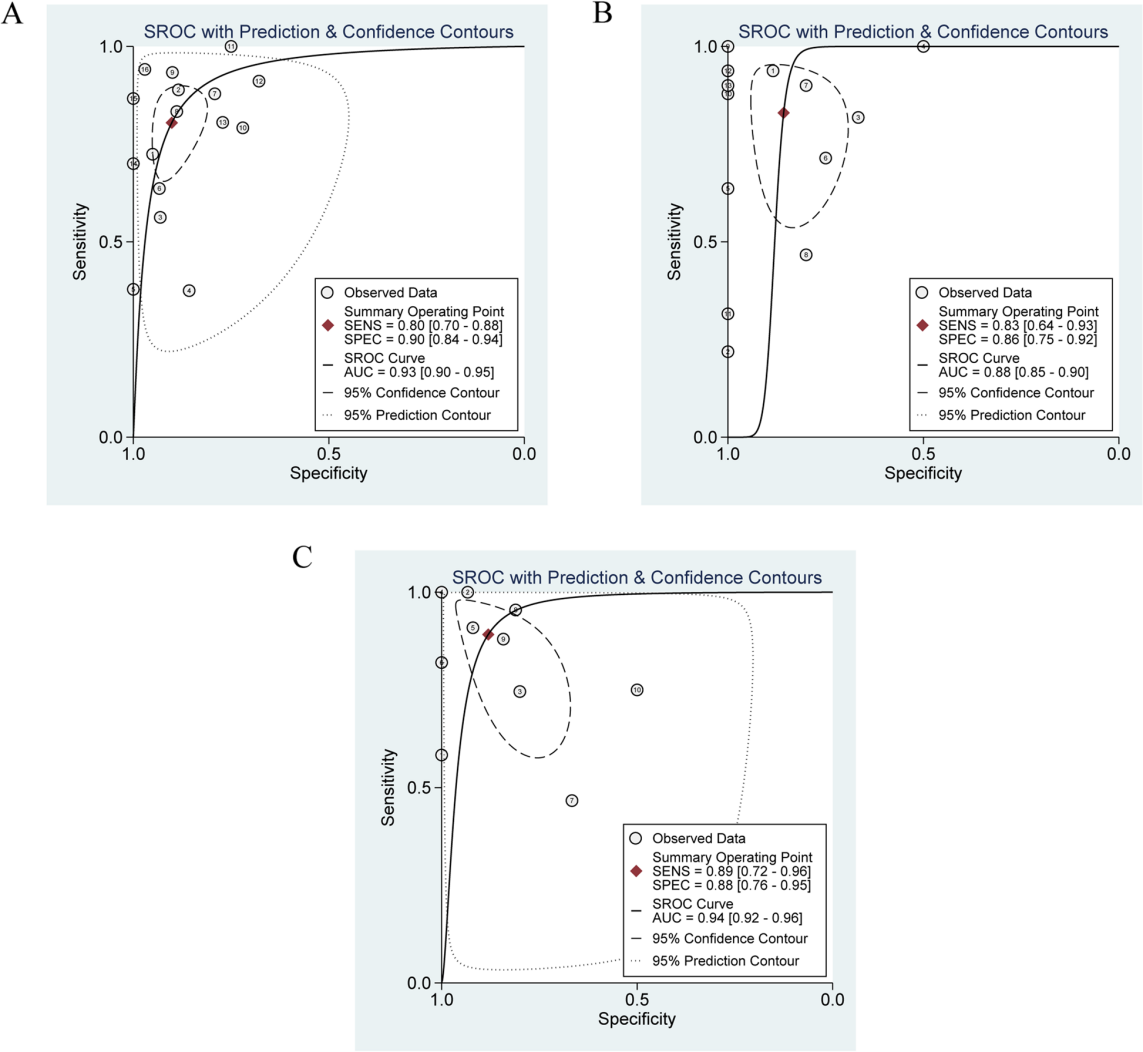
SROC curves for diagnostic performance of MRI, ^18^F-FDG PET and ^18^F-FDG PET/CT. A: SROC curve for diagnostic performance of MRI. B: SROC curve for diagnostic performance of ^18^F-FDG PET. C: SROC curve for diagnostic performance of ^18^F-FDG PET/CT

The numbers of SPECT or SPECT/CT studies utilizing ^99m^Tc-EC (ethylenecysteine), ^111^In-Girentuximab, ^99m^Tc-sestamibi (^99m^Tc-MIBI), and ^99m^Tc-methylene diphosphonate (^99m^Tc-MDP) were 1, 1, 2, and 3, respectively. The sensitivities of these studies ranged from 0.29 to 0.95 and the specificities ranged from 0 to 0.94. The numbers of PET or PET/CT studies using ^18^F-FDG (^18^F-fluorodeoxyglucose), ^18^F-fluoride, ^124^I-girentuximab, and ^11^C-acetate as radiopharmaceuticals were 20, 1, 1, and 1, respectively. We performed the sensitivity analysis to assess the impacts of single study on the overall outcomes. No study was identified as outliers. The final numbers of studies in terms of the meta-analysis of MRI, ^18^F-FDG PET and ^18^F-FDG PET/CT were 16, 13, and 10, respectively. The pooled sensitivity of MRI, FDG PET and FDG PET/CT were 0.80 [0.70,0.88], 0.83 [0.64, 0.93] and 0.89 [0.72, 0.96], respectively. The overall specificities were 0.90 [0.84,0.94], 0.86 [0.75, 0.92] and 0.88 [0.76, 0.95] for MRI, FDG PET and FDG PET/CT. The AUC values of MRI, FDG PET and FDG PET/CT were 0.93 [0.90, 0.95], 0.88 [0.85, 0.90] and 0.94 [0.92, 0.96] (see Figs. 3, 4, 5 and 6).

### Subgroup analysis of the performance of MRI

The pooled sensitivity and specificity of MRI studies at 1.5 T were 0.86 [0.64, 0.96] and 0.94 [0.76, 0.99], respectively. The AUC of MRI studies at 1.5 T was 0.96 [0.94, 0.98]. With respect to prospective MRI studies, the pooled sensitivity, specificity and AUC were 0.88 [0.81, 0.93], 0.91 [0.71, 0.98] and 0.90 [0.88, 0.93]. In the detection of primary RCC, MRI studies revealed a pooled sensitivity, specificity, and AUC of 0.76 [0.65, 0.85], 0.88 [0.81, 0.93], and 0.90 [0.87, 0.92] respectively. More details were shown in Table 2.

**Table 2:** Results of Subgroup analysis

Subgroups	Sensitivity	I^**2**^ of sensitivity (%)	Q value of sensitivity	p of sensitivity	Specificity	I^**2**^ of specificity (%)	Q value of specificity	p of specificity	PLR	NLR	DOR	SROC Curve AUC
**MRI at 1.5 T**	0.86 [0.64, 0.96]	92.11	63.39	< 0.001	0.94 [0.76, 0.99]	75.77	20.63	< 0.001	15.2 [3.3, 70.0]	0.15 [0.05, 0.42]	104 [18, 604]	0.96 [0.94, 0.98]
**Prospective studies**												
MRI	0.88 [0.81, 0.93]	26.43	5.44	0.25	0.91 [0.71, 0.98]	80.70	20.72	< 0.001	10.2 [2.7, 38.4]	0.13 [0.08, 0.22]	79 [15, 403]	0.90 [0.88, 0.93]
PET	0.90 [0.56, 0.98]	96.19	157.33	< 0.001	0.93 [0.54, 0.99]	64.60	16.95	0.01	12.6 [1.4, 117.3]	0.11 [0.02, 0.63]	119 [5, 2597]	0.97 [0.95, 0.98]
**Primary RCC**												
MRI	0.76 [0.65, 0.85]	79.90	59.70	< 0.001	0.88 [0.81, 0.93]	66.50	35.82	< 0.001	6.4 [4.3, 9.7]	0.27 [0.18, 0.39]	24 [16, 37]	0.90 [0.87, 0.92]
PET	0.77 [0.55, 0.91]	89.86	88.73	< 0.001	0.80 [0.60, 0.91]	0	5.37	0.80	3.8 [1.8, 7.9]	0.28 [0.13, 0.61]	13 [4, 45]	0.84 [0.80, 0.87]
PET/CT	0.80 [0.64, 0.90]	82.35	28.33	< 0.001	0.85 [0.73, 0.93]	20.38	6.28	0.28	5.5 [2.8, 10.8]	0.23 [0.11, 0.46]	24 [7, 78]	0.89 [0.85, 0.91]

*RCC* renal cell carcinoma, *MRI* magnetic resonance imaging, *PET* positron emission tomography, *CT* computerized tomography, *PLR* positive likelihood ratio, *NLR* negative likelihood ratio, *DOR* diagnostic odds ratio, *SROC* summary receiver operating characteristic, *AUC* area under the SROC curve

### Subgroup analysis of the performance of PET and PET/CT

With respect to prospective PET studies, the pooled sensitivity, specificity and AUC were 0.90 [0.56, 0.98], 0.93 [0.54, 0.99] and 0.97 [0.95, 0.98], respectively. In the detection of primary RCC, PET studies revealed a pooled sensitivity, specificity, and AUC of 0.77 [0.55, 0.91], 0.80 [0.60, 0.91], and 0.84 [0.80, 0.87], respectively. In addition, the pooled sensitivity, specificity, and AUC of PET/CT studies in detecting primary RCC were 0.80 [0.64, 0.90], 0.85 [0.73, 0.93], and 0.89 [0.85, 0.91], respectively. More details were shown in Table 2.

### Heterogeneity and publication bias

Deek’s tests for publication bias yielded *p* values of 0.94, 0.02, and 0.08 for MRI, FDG PET, and FDG PET/CT, which revealed that there was a possible publication bias in the pooled analysis of FDG PET studies.

## Discussion

Renal cell carcinoma is the most commonly diagnosed subtype of kidney cancers and accounts for approximately 2–3% of all malignancies [21]. The research of Motzer et al. demonstrated that the average 5-year survival rates for patients with RCC decreased with the disease stages (I to IV), from 96 to 23% [31]. Moreover, the early signs and symptoms of RRC are not specific which introduces difficulties for the early detection of this disease in primary or metastatic sites [32]. Renal biopsy is an accurate method to establish a histological diagnosis for RRC, however, it is may induce a risk of procedural adverse events [33]. Noninvasive approaches namely MRI, SPECT, and PET have been in evolution during the past decades [15, 34, 35]. Based on various studies of the diagnostic value of noninvasive modalities in the detection of RCC, we carry out a meta-analysis to compare the diagnostic efficacy of these approaches.

The meta-analysis was processed on the basis of study design, type of imaging modalities, type of radiotracers, type of RCC. To our knowledge, some of these dimensions have not been discussed in relevant meta-analyses [36–38]. Results revealed that the pooled sensitivity of PET/CT (0.89 [0.72, 0.96]) was the highest. MRI demonstrated the highest overall specificity (0.90 [0.84,0.94]). MRI and PET/CT showed high diagnostic performance in detecting RCC. Results of subgroup analysis manifested that PET/CT imaging had better performance than PET. Sensitivity and specificity of PET-CT are higher compared with PET alone. Furthermore, our research indicated that PET/CT and MRI revealed better performance in detecting primary RCC compared with PET alone. Due to the limited number of studies regarding recurrent or metastatic RCC, we didn’t conduct meta-analysis of this subgroup, this is one of the limitations of our study.

In this meta-analysis, we conducted a detailed literature search to improve the probability of searching as many related studies as possible. Two independent investigators completed the whole process of data extraction using standardized electronic forms. Furthermore, we evaluated the heterogeneity between the studies included. There were significant heterogeneities among studies. Distinctions in the year of publication, study methodology, patient characteristics, reference standard, and radiotracers may be the source of heterogeneity. Unfortunately, meta regression was not able to be performed to investigate the likely cause of heterogeneity due to limited number of covariates extracted from the enrolled studies. Subgroup analysis was undertaken to explore the possible source of heterogeneity. For the analysis of PET, the source of heterogeneity may be attributed to the type of radiotracers, type of RCC, and study design. However, not all potential source of heterogeneity was analyzed because of the insufficient number of studies in different subgroups. On account of this limitation, the efficacy of heterogeneity assessment in the study may be biased. Besides, publication bias was detected through the Deeks’ funnel plot asymmetry test in the analysis of PET studies. The publication bias may be attributed to the strict exclusion criteria of this meta-analysis. Although there is heterogeneity among studies included and publication bias, the findings of this analysis may introduce evidence and assistances concerning scientific research and clinical practice in the detection of RCC. In regard to further research, novel radiotracers with higher uptake ratios between tumor tissues to normal tissues and lower levels of renal excretion need to be further investigated on account of the results of this meta-analysis. In terms of application in the clinical setting, MRI is recommended as the favorable imaging method to help detect RCC due to lack of radiation exposure and high soft-tissue resolution. PET/CT shows better performance than PET alone for the diagnosis of RCC under the current development of functional imaging modalities. Of note, combined employment of various detection techniques is may be of assistance to increase the overall diagnostic accuracy. Interestingly, the hybrid PET/MRI, which provides combined anatomical and metabolic information, has drawn much attention in recent years, results of the study of PET/MRI in the detection of RCC is promising, and recent prospective studies are in progress [39, 40].

## Data Availability

The datasets used and/or analyzed during the current study available from the corresponding author on reasonable request.

## Abbreviations

RCC: Renal cell carcinoma
MRI: Magnetic resonance imaging
DW: Diffusion-weighted
SPECT: Single photon emission computed tomography
PET: Positron emission tomography
CT: Computerized tomography
PRISMA: Preferred Reporting Items for Systematic Reviews and Meta-analysis
TP: True positive
FP: False positive
TN: True negative
FN: False negative
PLR: Positive likelihood ratio
NLR: Negative likelihood ratio
DOR: Diagnostic odds ratio
CI: Confidence interval
SROC: Summary receiver operating characteristic
AUC: Area under the SROC curve
QUADAS-2: Quality Assessment of Diagnostic Accuracy Studies-2
